# 

**Published:** 1904-11

**Authors:** 


***NOTICE.—This JOURNAL and THE INTERNATIONAL JOURNAL OF SURGERY one year for $1.00 cash, to new subscribers. Out-of-town checks not accepted unless 10c. is added for cost of collection. Strictly to new subscribers.***

**This Journal and Atlanta Semiweekly Journal one year for $1.25 cash in advance. No checks. Strictly to new subscribers.**

